# Supplementary material for: Tap Water Consumption Is Associated with Schoolchildren’s Cognitive Deficits in Afghanistan
Source: Int J Environ Res Public Health. 2022 Jul 6;19(14):8252. doi: 10.3390/ijerph19148252 (PMC9321136; doi:10.3390/ijerph19148252)
Supplement: Supplementary file 1 [file ijerph-19-08252-s001.zip › supplement File S1.pdf]

POVERTY INDEX			
Read to respondent: "I would like to ask you some questions about your living conditions. I realize some of these questions seem unrelated to healthcare, but all of these questions help us to understand what our clients' living situations are like. This understanding helps us to plan services that people can easily access and more readily afford. Please answer as honestly as possible, as this will allow us to better serve the community. ***Your answers will not affect the service you receive or the price you pay****"* *Read questions to respondent exactly as written. Do not read out the response options. Circle the number corresponding closest to the respondent's answer. All questions must be answered.*			
P1	How many household members are 16-years-old or younger?	Seven or more....................................1<br>Five or Six...2<br>Four..3<br>Three..4<br>Two...5<br>One...6<br>None...7	
P2	Can both the male head/spouse and the female head/spouse read and write?	No male head/spouse..........................1<br>No female head/spouse.......................2<br>No ....3<br>Yes 4	
P3	What type of dwelling best describes where the household lives?	Temporary shelter/shack, part of a shared house, separate apartment, shared apartment, tent, or other..............................1<br>Single-family house...2	
P4	How many rooms (both exclusively yours and shared) does your household occupy (exclude corridors and balconies)?	One to four...1<br>Five or more...2	
P5	Which main toilet facility does the household use?	None (open field, bush) or sahrahi, dearan (area inside or outside compound but not pit), or other...1<br>Open pit...2<br>Traditional covered latrine.............................3<br>Improved latrine, or flush latrine..................4	
P6	In the past 30 days, what has been the household's main source of cooking fuel?	Animal dung, scavenged material/trash, bushes (ping)/twigs, branches, or other...1<br>Crop residues, firewood, charcoal/coal, kerosene or oil, gas, or electricity 2	

P7	How many stoves/gas cylinders does the household own?	None...1<br>One..2<br>Two or more 3	
P8	Does the household own any sewing machines?	No..1<br>Yes...2	
P9	Does the household own any motorcycles or cars?	No..1<br>Motorcycle only ....2<br>Car (regardless of motorcycle) 3	
P10	Did anyone in the household own or have access to any irrigated land in the most recent summer cultivation season, excluding a garden plot?	No..1<br>Yes...2	

Guidelines for Poverty Assessment in MSI

This document introduces the poverty assessment approach used by MSI and takes you through the steps for implementing it in your country. If you are measuring poverty in an Exit Interview, please also refer to the latest Exit Interview package available on the Best Practice Gateway: https://bestpractice.mariestopes.org/ and search for 'Exit Interview'.

Caution for printing this document:

This document contains the PPI questions for **all** MSI countries. Please do not print the whole document. Refer to Appendix 1 or 2 to select the poverty questions and indicator commentaries relating to your country.

1.0 Why measure poverty?

MSI aims to provide services to the poor and the underserved and, as an evidence-based organisation, we need to measure whether or not we are achieving this objective. In particular, certain service delivery channels, such as our mobile outreach model, are specifically designed to reach the poor – are they achieving this goal?

Poverty is a complex concept and can be measured with diverse approaches. The goal for MSI was to adopt a measurement approach that was accurate, while also being quick and easy to implement, analyse and interpret. While there are many different elements

of poverty (including social exclusion), measuring all of these is beyond the scope of most of the surveys we conduct at MSI.

MSI selected the **'Progress out of Poverty Index' (PPI)** to assess the proportion of its clients who are 'poor'. PPI results allow for comparisons across service delivery channels, countries, and organisations as well as aggregation at a global level. Because this tool is not available in all countries in which MSI operates, we offer a second-best tool, called the '**Multi-dimensional Poverty Index' (MPI)**, if the PPI is not available in your country. Please refer to Table 1 below to see which tool you should use in your country.

2.0 Uses of the poverty results

Possible uses of the poverty results include:

- Making evidence-informed programme decisions concerning strategies to reach the poor.
- Measuring the success or need for improvement of pro-poor interventions such as outreach activities and voucher programmes. Such interventions may have specific poverty targets against which the results can be measured. (This would however require enough sample size for the specific channel, facility, or project whose poverty result you would want to analyse. Refer to the section on sampling in the Exit Interview protocol for guidance on appropriate sample size and sample selection.)
- Computing High Impact CYPs (HICs). The proportion of clients living below the extreme poverty line of $1.25/day or proportion of MPI poor clients is one of the metrics for estimating HICs.
- Quantifying equity: Poverty is way of measuring equity which is one of MSI's core output indicators.
- Reporting to donors to demonstrate practically how we are reaching the poor and underserved.
- Aid pricing decisions. Knowing the percentage of clients in a particular delivery channel or facility who are poor can assist management to make decisions on price reviews and whether to charge subsidized prices or pursue full cost recovery. For instance, if a programme finds out that only few clients are poor, it may consider introducing measures to effectively target the poor, for example by subsidising costs. Alternatively, if a population based survey finds that a high proportion of potential clients are poor, this might guide a programme's pricing or marketing strategies.

For tips on how to ensure effective evidence use in your programme, refer to the 'Evidence to Action' section of the M&E manual, available on the Best Practice Gateway.

3.0 Understanding the Poverty Assessment Tools

3.1 Progress out of Poverty Index

The PPI was externally developed by Grameen Foundation and consists of a set of 10 country-specific indicators covering household characteristics, such as asset ownership, which help to estimate the likelihood of a household living below or above one or more international and/or national poverty lines. The PPI uses the World Bank's international poverty lines such as the $1.25 /day (purchasing-power parity (PPP)), $2.50/day (PPP) as well as other poverty lines such as the USAID extreme poverty line and national poverty line of each country.

What is $1.25/day PPP

The $1.25/day (Purchasing Power Parity) is a way to compare levels of poverty internationally. Analysts determined what could be bought in the US market for $1.25 and then calculated how much that same 'basket of goods' would cost in different countries. This amount is referred to as $1.25/day Purchasing Power Parity because it is how much you would need to buy the same basket of goods in each country - it's not actually $1.25. In some countries $1.25 would go a long way! The World Bank decided that anyone living on less than $1.25/day (PPP) has less than the basic amount a person needs to live, and so is in extreme poverty. The PPP measure is an important measure of individual-level poverty because it takes into account cost of living, unlike looking at a country's GDP.

The questions, responses, and weights for the PPI were derived from each country's most recent national household expenditure or income survey. The ten PPI questions were based on a balance of the following criteria:

- The question has a strong correlation with poverty, i.e. there is statistical significance that households who answered the question in a certain way are below the poverty line.
  Example: "What is the level of education attained by the head of the household?"
- The question is inexpensive to collect, easy to answer quickly, and simple to verify.
  Example: "Of what material is the roof of the residence made?"
- The question is liable to change over time as poverty level changes.
  Example: "Does the household own a motorbike or car?

The outcome is a set of 10 country-specific questions that strongly predict a household's likelihood of being below a poverty line yet that can be easily implemented.

<u>PPI questions and responses cannot be changed – they must be asked exactly as they are in the scorecard.</u> The questions have been selected based on statistical

analysis and even small changes will make the analysis invalid. Also, it is important to understand that none of the 10 questions asked is analysed individually. The individual indicators combine to give the likelihood of that individual living below a poverty line. This means that **<u>it is important to ask each question of each respondent as it is not possible to obtain the poverty result for a respondent who does not answer all the poverty questions.</u>**

A full description of the PPI methodology is available at www.progressoutofpoverty.org.

Understanding PPI results

The PPI can be used to estimate 'poverty likelihood' of a particular household; that is, the probability that the household lives below a given poverty line. In addition, by averaging the overall likelihood scores, programmes can estimate the proportion of their clients living below a given poverty line.

MSI has adopted the World Bank definition of extreme poverty – living under $1.25/day (PPP) as the standard measure of 'poor clients', because it is an internationally recognised standard and allows for comparison of our results between countries and with other organisations. In addition to the standard poverty line adopted by MSI, a country programme that is interested in knowing the proportion of its clients living below $2.50/day (PPP) can do that analysis with the same data. This poverty line is particularly suitable for 'middle-income' countries that are likely to have fewer clients living in extreme poverty. The PPI also allows country programmes interested in their national poverty lines to calculate estimates of the proportion of the target population living under those.

3.2 The Multi-dimensional Poverty Index

Unfortunately the PPI is not available in all MSI countries. For those countries where the PPI has not yet been developed, we use the Multi-dimensional Poverty Index (MPI). The MPI was externally developed by Oxford Poverty and Human Development Initiative (OPHI) and has a set of 14 questions covering three main areas of deprivation - health, education and living standards.

MPI Indicators are not country specific, meaning the same indicators are used for all countries. Three main datasets were used to derive the MPI - the Demographic and Health Survey (DHS), the Multiple Indicators Cluster Survey (MICS), and the World Health Survey (WHS). Indicators were based on the most recent and reliable data available.

MPI questions and responses cannot be changed – they must be asked exactly as they are in the scorecard. The questions have been selected based on statistical analysis and even small changes will make the analysis invalid. Additionally, it is important to understand that none of the 14 questions asked is analysed individually but the indicators are combined together to give the single output of whether or not the individual is poor. This means that **it is important to ask each question of each respondent as it is not possible to obtain the poverty result for a respondent who does not answer all the poverty questions.**

A full description of the MPI methodology is available at http://www.ophi.org.uk

Understanding MPI results

The MPI does not give us the proportion of clients living below a given poverty line such as $1.25/day or the national poverty line, but identifies households as 'Multi-dimensionally poor', taking into consideration education levels, health and living standards. As defined by OPHI, a household that is Multi-dimensionally poor is said to live in 'acute poverty'. The list of MPI questions is very similar to those used in the PPI and also allows for comparisons of the proportion of poor clients across service delivery channels and over time.

4.0 How to Implement the Poverty Assessment Tools

The PPI/MPI can be included in any survey. We recommend adding these questions towards the end of the questionnaire as some of the questions may be considered sensitive.

- To find out the proportion of <u>your clients</u> who are poor, add the PPI/MPI to your Exit Interviews.
- To find out the proportion of <u>a wider population</u> who are poor, add the PPI/MPI to a community-level survey.

Any questionnaire that the PPI/MPI is added to should also include the following key socio-demographic questions: respondent's age; gender; education level; marital status; occupation; number of children. This will help to give the full picture of your clients' profile and a deeper understanding of the poverty results.

For detailed guidance on conducting an Exit Interview see the Exit Interview Package 2013 at: https://bestpractice.mariestopes.org/ and contact your RME Advisor.

To find out the right set of questions for your country, refer to Table S1 below.

Table S1. Countries with PPI and those with MPI.

These countries should use the **PPI**:[1]		These countries should use **MPI**:[3]
Afghanistan	Nepal	Madagascar
Bangladesh	Nigeria	Mongolia
Bolivia	Pakistan	PNG
Burkina Faso	Philippines	South Sudan
Cambodia	Sierra Leone	Zimbabwe
Ethiopia	Senegal	China (contact RME)
Ghana	South Africa	
India	Sri Lanka	
Kenya	Tanzania	
Malawi	Timor Leste	
Mali	Uganda	
Mexico	Viet Nam	
Myanmar	Yemen	
	Zambia	

GO TO APPENDIX 2 FOR THE TOOL

GO TO APPENDIX 1 FOR THE TOOL

Once you have identified which tool is right for your country, please find the correct tool in the relevant Appendix. If your country has a PPI, there is a different set of questions for each country - be sure to use the correct one! If you are using the MPI, there is only one set of questions.

Note that for some countries PPI translations are available. The links can be found in Appendix 2 below.

[1] Correct as of May 2013

4.1 Administering PPI/MPI questions

The following are guidelines for conducting the MPI/PPI interviews. We recommend including these in your interviewer/enumerator training.

- Strictly adhere to the questions and response options. Do not modify or vary the questions' meaning in any way.
- Follow the definitions and guidelines for interpreting the questions, which are based on the national surveys' enumerator manual. Please refer to Appendix 2 for the commentaries pertaining to your country's PPI.
- Encourage respondents to answer every question. If a respondent fails to answer any of the poverty questions, the overall poverty likelihood for that respondent cannot be computed. Interviewers **should not**, however, force respondents to answer questions they are not willing to.
- If at any point the client expresses some discomfort with the interview, the interviewer should return to a more informal dialogue to make the client feel at ease, before resuming the interview.

The poverty questions, wording, answer categories and the response codes cannot be changed. Do not modify or vary the questions in any way as a slight change could make the result invalid.

4.2 Ensuring Data Quality in Poverty Assessment

Before administering the PPI/MPI, the questionnaire must be translated into any relevant local languages. (The links to some standard local language translations for the PPI are available in Appendix 2)

The following are some tips to ensure high quality translations.

- Pay a professional interpreter to translate the questionnaire
- Translate into local language, and back-translate into English
- If possible, two interpreters translate and then work together to agree best wording
- Interpreters should always focus on the meaning, not word-for-word translation
- Translation should never be done by the interviewer, but always in advance

In training interviewers, follow these recommendations to ensure data quality:

- Interviewers should be trained in a classroom setting
- Let interviewers practice interviews in role-plays
- Observe interviewers in on-site monitoring visits
- Work through the whole questionnaire with interviewers and teach how to:
    - follow good informed consent practices (non-coercion, interviewers should allow the participant as much time as they want to decide to participate)
    - select respondents following your sampling strategy
    - administer the questionnaire – don't lead, don't change the wording, and

- mark responses correctly; e.g. the number corresponding to the response given should be *circled* (not ticked, not crossed etc.). Do not leave any question blank.

- Double data entry is recommended to assure accurate entry and to check for inconsistency among data entry clerks.

5.0 How to analyse the Poverty Assessment data

The Evidence, Strategy and Innovation Team have produced a data entry template and analysis tool to help you analyse the poverty data. Please contact your RME Advisor for these tools or visit https://bestpractice.mariestopes.org to download them.

For countries using the PPI

Once the data have been entered in the standard data entry template (keeping all categories and codes the same), simply run the poverty syntax provided as part of the latest Exit Interview Package. The poverty syntax computes: the poverty score for each individual; the likelihood of that individual being below the poverty line ($1.25, $2.50 and national); and, ultimately, the proportion of clients classified as poor (according to the different poverty lines). The syntax also analyses poverty results for each service delivery channel separately. This is useful because we would expect our outreach to target poorer women than our clinics. Below is an example of the output you will get in SPSS and how to interpret it. Example for MSI Fixed Centre:

Descriptives

	Type of Facility			Statistic	Std. Error
Likelihood respondent is below the $1.25 poverty line	MSI Fixed Centre	Mean		.217	.00274
		95% Confidence Interval for Mean	Lower Bound	.0163	
			Upper Bound	.0270	
		Std. Deviation		.04132	
		Minimum		.00	
		Maximum		.34	
Likelihood respondent is below the national poverty line	MSI Fixed Centre	Mean		.283	.00353
		95% Confidence Interval for Mean	Lower Bound	.0214	
			Upper Bound	.0353	
		Std. Deviation		.05325	
		Minimum		.00	
		Maximum		.40	

The figure we are particularly interested in here is the **mean**. To report this as a percentage simply multiply by 100. This tells us the proportion of our clients who fall below the specified poverty lines ($1.25 and national in the example above). Your interpretation will be:

In the MSI Fixed Centres in [country], about 22% of clients were found to be living below the $1.25 poverty line while about 28% were living below the national poverty line.

Poverty figures obtained from our surveys are estimates and should be approximated to the nearest whole number when reporting them as demonstrated above.

For countries using the MPI:

Once the data have been entered (keeping all categories and codes the same), simply run the syntax provided. This will compute the proportion of clients classified as 'Multi-dimensionally poor'. Below is an example of the output you will get in SPSS and how to interpret it. Example for MSI Fixed Centre.

MPIpov MPI poverty outcome

		Frequency	Percent	Valid Percent	Cumulative Percent
Valid	.00 not poor	103	64.4	66.9	66.9
	1.00 poor	51	31.9	33.1	100.0
	Total	154	96.2	100.0	
Missing	System	6	3.8		
Total		160	100.0		

The figure we are interested in here is the **valid percent** that is in category 1 Poor. Your interpretation will be:

In the MSI Fixed Centres in [country], 33% of clients were found to be 'Multi-dimensionally poor' according to the OPHI Multi-dimensional Poverty Index.

Poverty figures obtained from our surveys are estimates and so should be approximated to the nearest whole number when reporting them as demonstrated above.

5.1 Comparison with National Data

One way to assess how well you are reaching the poor in your country is to compare the poverty results from your Exit Interview with the same poverty results for the national population. For instance, you may compare the percentage of your outreach clients living in extreme poverty to the percentage of the national population living in extreme poverty. If the percentage of your clients that are poor is significantly greater than the percentage of the national population that is poor, it suggests that you have been effective in reaching the poor. In other words, your clients are, on average, poorer than the national population and you are successfully targeting the poor within your country. The reverse is true if the percentage of your poor clients is significantly lower than the percentage of the poor in the national population.

For you to say there is a significant difference between the percentage of your clients who are poor and the percentage of the national population living in poverty, there should be a difference of at least ±10 percentage points. Thus, if the difference is less than ±10 percentage point, it means that the percentage of your clients that are poor is approximately the same as the national population.

Example: If 54% of your outreach clients were found to be poor compared to 32% of the poor in the national population, then a greater proportion of your outreach clients are poor than the national population. This would imply that your programme is successfully targeting the poor. If 38% of your outreach clients were found to be poor, compared to 32% of the population, then we cannot be sure if there is a difference. We can only conclude that the outreach clients are broadly similar to the national population. However, if 20% of your outreach clients were found to be poor compared to the 32% nationally, this would imply that you have not be effective in targeting the poor in your country.

5.2 Interpretation of poverty results

What does it mean to say x% of clients live below $1.25/day

The World Bank determines that anyone living on less than $1.25/day (PPP) has less than the basic amount a person needs to live, and so is in extreme poverty. Below is an example of the profile of people living below the extreme poverty line as analysed by Abhijit Banerjee and Esther Duflo (2006)[2] of the Department of Economics at Massachusetts Institute of Technology (MIT).

---

[2] Banerjee, Abhijit V. and Duflo, Esther, The Economic Lives of the Poor (October 31, 2006). MIT Department of Economics Working Paper No. 06-29. Available at SSRN: http://ssrn.com/abstract=942062 or http://dx.doi.org/10.2139/ssrn.942062

- The typical extremely poor family tends to be rather large, at least by the standards of today's rich countries. The number of family members varies between about 6 and about 12, with a median value (across the different countries) of between 7 and 8
- **About 55-80% of their income is spent on food. Among the extremely poor, only 57 percent report that the members of their household had enough to eat throughout the year.**
- **The extremely poor spend very little on education. The expenditure on education generally hovers around 2 percent of household budgets: higher in Pakistan (3 percent), but much lower in South Africa (0.8 percent).**
- People living in extreme poverty generally reported owning few "productive" assets such as bicycles, sewing machines, phones, or tractors, though in some areas a large proportion of poor households own small plots of land.
- The poor often lacked access to basic infrastructure and, as with assets, there was large variability among households around the world. In Mexico and Indonesia, for example, electricity access was nearly universal, but in-house tap water and ownership of a toilet or latrine were far less prevalent in Indonesia. In Tanzania, the pattern was quite different: nearly every poor household owned a toilet and very few had access to electricity or in-house tap water.

What does it mean to say x% clients are MPI poor

An individual who suffers deprivation in at least 30% of the 14 weighted MPI indicators is considered to be MPI poor or is deemed to suffer from 'acute poverty'. Multi-dimensional poverty combines several factors that constitute poor people's experience of deprivation – such as poor health, lack of education and inadequate living standard.

6.0 Strengths and Limitations

Strengths of PPI/MPI

- It is easy to add PPI/MPI questions to questionnaires for other surveys such as Exit Interview, baselines studies etc.
- PPI/MPI questions are easy to administer within a short time
- PPI/MPI data is easy to analyse and to interpret results
- PPI uses international poverty lines adjusted for purchase power parity which makes it possible to compare results across service delivery channels and countries.
- The fact that PPI and MPI have multiple indicators which helps to estimate poverty helps to capture the multiple dimensions of poverty beyond just household income.
- The PPI/MPI provides more accurate poverty assessment than proxies such as education level or occupation.

Limitations of PPI/MPI

- The PPI does not tell whether an individual client is definitely below the poverty line or not. At the individual level it can only show how likely it is that that person is below a poverty line. This also means that the analysis we can do at the individual level is limited.
- The MPI does not allow us to measure the proportion of our clients living below an internationally accepted poverty line. It only tells the proportions of clients who are Multi-dimensionally poor.
- If implemented through MSI's Exit Interviews using the standard sample size guidelines, the small sample sizes also mean that sub-group analysis is not possible for both PPI and MPI.

7.0 Frequently Asked Questions about the Poverty Index

Even though a house is built of iron/concrete or tiles, the household members may be living on less than $1.25.

That's true of course. From an Exit Interview we cannot say for certain if each individual is poor or not – that would require too many questions. By looking at the total score from all 10 questions (not at each question individually) this tool gives us the 'likelihood' of an individual being poor. Then the aggregate score will tell us the proportion of our clients who are poor.

Why not just ask household income?

We generally find that asking directly about an individual's household income will give very high levels of non-response. In addition, it's possible that some clients will not know what their household income is, or might have such irregular income that it is difficult to answer. Finally, the $1.25 poverty line is based on more than just household income.

These questions don't work in my country.

Where the Progress out of Poverty Index tool is available, each country has a set of questions developed specifically for that country, based on a high quality, nationally representative expenditure survey. This means that all the questions and categories have been developed for and tested in your country. Please stick with the questions as they are, even small changes will make the analysis invalid. As always, you may insert additional questions at the end of the Exit Interview questionnaire, if you think it's necessary but those questions should be analysed separately.

Appendix 1: Multi-dimensional Poverty Index

The following questions should be inserted into the end of the Exit Interview questionnaire, for countries where the Progress out of Poverty Index (Appendix 2) is not available. The questions, categories and codes must not be changed. At question P2 enter the normal ages for grades 1-8 in your country (i.e. not taking into account pupils re-sitting years).

**MULTI-DIMENSIONAL POVERTY INDEX**			
P1	Have any household members completed 5 years of more of schooling?	Yes..1<br>No..……………..0	
P2	In your household, are there any children aged x to y (grades 1-8) who are **not** attending school now?	Yes…………..1<br>No…..0	
P3	Has a child aged under 5 in your household died?	Yes………..1<br>No…..0	
P4	Has anyone in your household been tested for malnutrition?	Yes………..1<br>No…..0	→ P6
P5	If yes, did any of them test positive?	Yes………..1<br>No…..0	
P6	Do you have electricity at home?	Yes…………..1<br>No…..0	
P7	How far from your house is the nearest source of safe drinking water?	Less than a 30 minute walk……..................1<br>More than a 30 minute walk or no access to safe drinking water 0	
P8	Do you use a toilet or latrine?	Yes…………..1<br>No…..0	→P11
P9	Is the toilet or latrine shared with other households?	Yes…………..1<br>No…..0	→P11
P10	What type of toilet or latrine do you use?	Pit latrine with slab or ventilated improved latrine or flush/pour flush to sewer/septic tank /pit latrine………………………………1Other…..<br>...0	

Continues on following page!

P11	What is the floor made of in your home?	Dirt, sand or dung ……………………………..1<br>Other…………………………………………...0	
P12	What fuel do you mainly use for cooking?	Wood, charcoal or dung..........................1<br>Other…………………………………………...0	
P13	Does your household own a car or tractor?	Yes……………………………………………...1<br>No………………………………………………0	→end
P14	How many of the following does your household possess: Telephone, radio, TV, bicycle, motorbike, and fridge.	More than 1. ....1<br>1 or none...... 0	
